# Comparison of Diagnostic Criteria for Severe Ischemic Stroke: A Retrospective Cohort Study

**DOI:** 10.1002/brb3.71620

**Published:** 2026-07-23

**Authors:** Xiaoyu Hua, Jinzhen Song, Chengfeng Xing, Chenchen Wei

**Affiliations:** ^1^ Department of Neurology The Affiliated Hospital of Qingdao University Qingdao China; ^2^ Department of Abdominal Ultrasound The Affiliated Hospital of Qingdao University Qingdao China

**Keywords:** diagnostic ability, diagnostic criteria, National Institutes of Health Stroke Scale, severe ischemic stroke

## Abstract

**Background:**

The diagnostic criteria for severe ischemic stroke (SIS) vary across studies. There are no generally accepted diagnostic criteria for SIS and no consensus on which criteria are most appropriate in clinical practice. We aimed to evaluate the clinical significance of these criteria by comparing their associations with death/disability in an independent clinical dataset.

**Methods:**

This was a retrospective cohort study that consecutively included ischemic stroke patients admitted within 7 days of onset. We conducted a literature review to determine the commonly used diagnostic criteria for SIS. We used the sensitivity, specificity, and Youden index to assess the relationship of the criteria with the poor outcomes and calculated the area under the receiver‐operator characteristics curve (AUC).

**Results:**

We identified 10 commonly used diagnostic criteria for SIS through literature review, including four clinical criteria, three imaging criteria, and three composite criteria. A NIHSS score ≥ 15 had the maximal Youden index (0.395) and AUC (0.697, 95% CI 0.641–0.754) for predicting poor outcomes at 3 months, followed by NIHSS 1a ≥ 1 (Youden index 0.387; AUC 0.693, 95% CI 0.637–0.750). Despite the poor comprehensive diagnostic ability, the composite criteria had a high specificity of over 97% and a negative predictive value of nearly 84%. For death or transfer to the intensive care unit during hospitalization, NIHSS 1a ≥ 1 had the maximal Youden index (0.596) and AUC (0.798, 95% CI 0.728–0.869), followed by NIHSS ≥15 (Youden index 0.539; AUC 0.769, 95% CI 0.692–0.847).

**Conclusions:**

The severity of stroke assessed by NIHSS ≥ 15 and the impaired consciousness defined by NIHSS 1a ≥ 1 showed the best diagnostic ability for SIS. Our results provide scientific evidence to identify a simple and unified diagnostic criterion for SIS, which will help to enable cross‐comparison between research and facilitate communication of case series in clinical practice.

## Graphical abstract

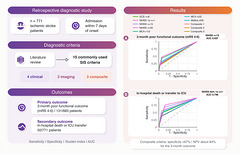

**Abbreviations**
AUC: area under the receiver‐operator characteristics curve
CI: confidence interval
CT: computed tomography
GCS: Glasgow Coma Score
MCA: middle cerebral artery
MRI: magnetic resonance imaging
mRS: modified Rankin scale
NIHSS: National Institutes of Health Stroke Scale
NPV: negative predictive value
PPV: positive predictive value
SIS: severe ischemic stroke

## 1 Introduction

Stroke remains a major global cause of death and disability. The GBD 2021 study estimated 93.8 million prevalent cases and 11.9 million incident cases worldwide in 2021 (GBD 2021 Stroke Risk Factor Collaborators 2024). The World Stroke Organization further estimated that the annual global cost of stroke exceeded $890 billion, underscoring its impact on patients, caregivers, and health care systems (Feigin et al. 2025). In China, this burden is also substantial. Ischemic stroke accounts for approximately four‐fifths of incident strokes in Chinese adults, and stroke‐related disability remains common, with disability rates of approximately 14%–15% among stroke survivors at 3–12 months after stroke (Wu et al. 2026). These findings further highlight the long‐term impact of acute ischemic stroke on functional independence, caregiver burden, rehabilitation demand, and health care resource use. Within this broader burden, severe ischemic stroke (SIS) represents a particularly devastating subgroup: the mortality of conservatively managed patients with SIS is approximately 80% (Hacke et al. 1996; Berrouschot et al. 1998), and most survivors have a persistent severe disability (Subramaniam and Hill 2005). More research on risk factors, pathophysiology, and management of SIS is needed. However, relevant research is precluded by the diversity in the terms defining SIS, such as malignant middle cerebral artery (MCA) infarction, space‐occupying ischemic stroke, massive ischemic stroke, and large hemispheric stroke. The criteria for diagnosing SIS also vary across studies. For example, clinical trials on hemicraniectomy in patients with SIS used different cut‐off points of the National Institutes of Health Stroke Scale (NIHSS) score as one of the inclusion criteria (Vahedi et al. 2007; Hofmeijer et al. 2009; Jüttler et al. 2007); some researchers used the ischemic extent involving > 50% of MCA territory or an infarct volume > 145 mL on the diffusion‐weighted imaging as a criterion for SIS (Vahedi et al. 2007), while others used the ischemic extent > 2/3 of MCA territory (Jüttler et al. 2014). Such variation in diagnostic criteria prevents cross‐study comparisons. Besides, there are no generally accepted diagnostic criteria for SIS and no consensus on which criteria are most appropriate in clinical practice.

We aimed to evaluate the clinical significance of the existing criteria for SIS by comparing their associations with short‐term death/disability in an independent clinical dataset. Rather than proposing another definition, this was the first study to comprehensively compare commonly used criteria derived from the preexisting evidence side by side in the same independent cohort and relate each criterion to clinically meaningful outcomes. The final definition identified in the study would contribute to early diagnosis and management of severe stroke in practice and facilitate the identification and inclusion of patients in clinical trials on SIS.

## 2 Methods

### 2.1 Selection of Criteria for SIS

We searched PubMed from 1952 to June 2024 with search terms “large hemispheric infarction,” “malignant middle cerebral artery infarction,” “cerebral infarction with swelling,” and “severe ischemic stroke.” We included clinical trials or systematic reviews that elaborated the clear criteria for defining stroke patients with a “severe” condition during the acute phase. We included studies published in English.

**FIGURE 1:**
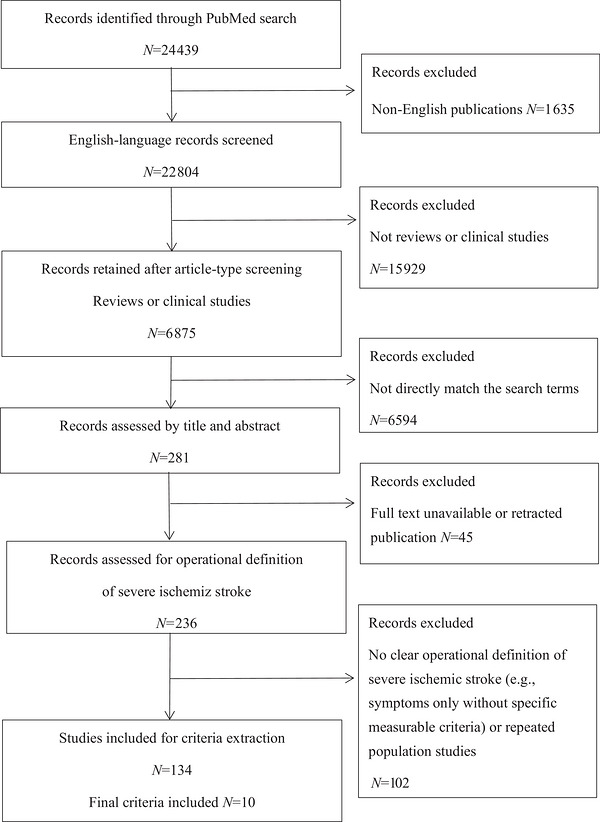
Flowchart of literature search and selection.

We first extracted the diagnostic criteria reported in the literature and then listed all components of the criteria and ranked each component according to their frequencies reported by the included studies. We only considered criteria that are suitable for quick bedside assessment without depending on potentially time‐consuming, computer‐based systems. The literature search and selection process is summarized in Figure 1.

### 2.2 Study Participants

This was a retrospective cohort study. Consecutive ischemic stroke patients admitted to the Department of Neurology, the Affiliated Hospital of Qingdao University, between January 1, 2021 and June 30, 2022 were included. Inclusion criteria were (1) age > 18 years; (2) a diagnosis of ischemic stroke; (3) admission within 7 days after stroke onset; and (4) brain computed tomography (CT) or magnetic resonance imaging (MRI) within 7 days after onset. Exclusion criteria were (1) preexisting significant disability defined by a modified Rankin scale (mRS) score ≥ 2; (2) severe heart failure, respiratory failure, liver, or renal dysfunction; and (3) other severe systemic diseases. The inclusion and exclusion criteria are summarized in Table S1.

The project was approved by the Biomedical Research Ethics Committee of the Affiliated Hospital of Qingdao University (No. QDFYWZLL‐20240114‐003), and the protocol conformed to local ethics criteria for human research. All participants provided the written informed consent. Although this was a retrospective cohort study, we organized the reporting of the diagnostic accuracy components according to the STARD 2015 reporting guideline (Supporting Information).

### 2.3 Data Collection

Demographic information (age and sex) and clinical data, including prior hypertension, diabetes mellitus, atrial fibrillation and hyperlipidemia, smoking and drinking history, time from onset to hospital, NIHSS score and Glasgow Coma Score (GCS) on admission, intravenous thrombolysis, and endovascular treatments, were extracted from the electronic medical records using a predefined standardized data extraction form. The endovascular treatments included mechanical thrombectomy, balloon angioplasty, or stenting.

The initial brain imaging (CT or MRI) was performed within 24 h after admission. Patients underwent repeated neuroimaging examinations based on the clinical arrangement, such as neurological worsening. All the images acquired from admission to 7 days after onset were evaluated and the extent of the ischemic region and the midline shift on neuroimaging were recorded. A midline shift was defined as a midline shift of > 5 mm at the septum pellucidum level or > 2 mm at the pineal gland level (Delgado et al. 2006). The clinical and imaging data used to determine the candidate SIS criteria were extracted from electronic medical records and neuroimaging data before assessment of the 3‐month mRS outcome. The assessors of the 3‐month mRS outcome were not involved in extracting the index‐test variables.

### 2.4 Outcome Assessment

The primary outcome was a poor functional outcome, as defined by a mRS score of 4–6, at 3 months. The mRS is a measure of disability, which ranges from 0 (*no symptoms*) to 5 (*severe disability and bedridden*); for statistical purposes, death was given a score of 6. The mRS was assessed by telephone interviews. The secondary outcome was in‐hospital death or transfer to the intensive care unit during hospitalization.

### 2.5 Sample Size Estimate

Sample size adequacy was assessed using the conventional events‐per‐variable rule for multivariable logistic regression. We hypothesized that each multivariable model included eight prespecified covariates and that each variable required at least 10 outcome events. Finally, at least 80 outcome events were required. Assuming an expected 3‐month poor functional outcome rate of 15% based on previous studies (Wu et al. 2026), at least 534 patients with 3‐month outcome data were required. After allowing for a 15% loss to follow‐up, the required number of eligible patients was 628.

### 2.6 Statistical Analysis

Normality of continuous variables was assessed using histograms, *Q–Q* plots, and the Shapiro–Wilk test. Normally distributed continuous variables are presented as mean (standard deviation), non‐normally distributed variables as median (interquartile range [IQR]), and categorical variables as number (%). Patients without 3‐month outcome data were excluded from the primary outcome analysis, and no imputation was performed. We first performed a univariate logistic regression analysis using each criterion as an independent variable and the outcomes as the dependent variable. Multivariable logistic regression models were then constructed separately for each diagnostic criterion and adjusted for covariates that were clinically relevant or showed a *p* value < 0.20 in univariable analyses. We then calculated the sensitivity, specificity, Youden index, positive likelihood ratio, negative likelihood ratio, positive predictive value (PPV), and negative predictive value (NPV) of the selected criteria against the prespecified outcomes. Sensitivity, specificity, PPV, and NPV were calculated from the corresponding 2 × 2 contingency tables, and 95% confidence intervals (CIs) were calculated using the Wilson method. Specifically, sensitivity was calculated as true positives/(true positives + false negatives) × 100%, specificity as true negatives/(true negatives + false positives) × 100%, PPV as true positives/(true positives + false positives) × 100%, and NPV as true negatives/(true negatives + false negatives) × 100%. The area under the receiver operating characteristic curve (AUC) and 95% CI were also used as measures of diagnostic performance. Student *t*, chi‐squared, and Mann–Whitney *U* tests were conducted as appropriate to compare the characteristics of patients in different groups. Statistical analyses were performed using SPSS version 26.0.

## 3 Results

### 3.1 The Pre‐existing Diagnostic Criteria for SIS

The diagnostic criteria for SIS usually include the clinical and imaging factors (Hofmeijer et al. 2009; Jüttler et al. 2014; Landreneau and Sheth 2017; Frank et al. 2014; Fan et al. 2021; Pallesen et al. 2019). The clinical factors include the symptoms referable to the large vessel occlusion, NIHSS score, and GCS score (Yuan et al. 2021; Flechsenhar et al. 2013; Kirkman et al. 2014). Patients with sudden large vessel occlusion typically show dense hemiplegia, head and eye deviation, unilateral severe hypesthesia or anesthesia, homonymous quadrantanopsia or hemianopsia, and either aphasia or neglect, depending on the hemisphere (Landreneau and Sheth 2017; Flechsenhar et al. 2013). The cut‐off point of the NIHSS score used to define SIS was from 7 to 25, while a score of > 15 in the NIHSS was more widely used in previous studies (Vahedi et al. 2007; Hofmeijer et al. 2009; Jüttler et al. 2014; Pallesen et al. 2019; Flechsenhar et al. 2013; Kirkman et al. 2014; Lee et al. 2010; Schönenberger et al. 2016; Klehmet et al. 2009; Broderick et al. 2015; Demaerschalk et al. 2016; Libman et al. 2000; Steiner and Brainin 2003; Deng et al. 2019; Kvistad et al. 2019; Shao et al. 2018). A decreased level of consciousness, which is determined by a GCS score ≤ 8 or a score of ≥ 1 in item 1a of the NIHSS, is another important clinical indicator of SIS (Flechsenhar et al. 2013; Kirkman et al. 2014; Vahedi et al. 2007). As for the imaging criteria for SIS, a large ischemic extent defined by an ischemic sign involving > 1/2 or > 2/3 of the MCA territory on CT and brain swelling defined by a midline shift are widely used in most studies (Hofmeijer et al. 2009; Jüttler et al. 2007; Frank et al. 2014; Zha et al. 2015; Kondziella et al. 2013). Several studies used a DWI infarct volume > 80 mL within 6 h of onset or > 145 mL within 14 h as an inclusion criterion of SIS patients (Vahedi et al. 2007; Zha et al. 2015; Mlynash et al. 2011; Ronchetti et al. 2014). Although MRI, particularly DWI, is becoming more common in the assessment of stroke, non‐contrast CT remains the first choice of neuroimaging examination in acute stroke (Landreneau and Sheth 2017). Finally, we identified 10 commonly used criteria for SIS, which included four clinical criteria (GCS score ≤ 8, NIHSS 1a ≥ 1, NIHSS score ≥ 15, and NIHSS score ≥ 20), three imaging criteria (the ischemic extent involving > 1/2 or > 2/3 of the MCA territory and midline shift), and three composite criteria (NIHSS > 15 + NIHSS1a ≥ 1 + ischemic extent > 1/2 of the MCA territory [Composite criteria 1], NIHSS ≥ 15 + NIHSS1a ≥ 1 + ischemic extent > 2/3 of the MCA territory [Composite criteria 2], and NIHSS > 15 + GCS ≤ 13 + ischemic extent > 2/3 of the MCA territory [Composite criteria 3]; Table 1).

**TABLE 1:** Description of the diagnostic criteria for severe ischemic stroke.

Item	Components
Clinical factors	GCS ≤ 8
	NIHSS 1a ≥ 1
	NIHSS ≥ 15
	NIHSS ≥ 20
Imaging factors	Ischemic extent > 1/2 of the MCA territory
	Ischemic extent > 2/3 of the MCA territory
	Midline shift
Composite criteria	NIHSS > 15, NIHSS 1a ≥ 1, ischemic extent > 1/2 of the MCA territory
	NIHSS ≥ 15, NIHSS 1a ≥ 1, ischemic extent > 2/3 of the MCA territory
	NIHSS > 15, GCS ≤ 13, ischemic extent > 2/3 of the MCA territory

Abbreviations: GCS, Glasgow Coma Scale; MCA, middle cerebral artery; NIHSS, National Institutes of Health Stroke Scale.

### 3.2 Patient Characteristics

A total of 771 patients were enrolled in this study, of whom 488 (63.3%) were men, and the mean age was 64.6 (14.3) years (Table 2, Figure S1). The median time from onset to hospital admission was 12 h (IQR 3–48 h). The median NIHSS score and GCS score on admission were 5 (IQR 2–12) and 15 (IQR 13–15), respectively. There were 95 patients (12.3%) presenting with an ischemic extent > 2/3 of the MCA territory and 121 (15.7%) with an ischemic extent > 1/2 of the MCA territory. The midline shift occurred in 72 patients (9.3%). Forty patients (5.2%) were treated with intravenous thrombolysis and 27 (3.5%) patients received endovascular treatment.

**TABLE 2:** Baseline characteristics of enrolled patients.

	All patients	Patients with mRs of 4–6	Patients with mRs of 0–3	*p* value^a^
*N*	771	131	552	—
Sex, male	488 (63.3)	64 (48.9)	368 (66.7)	< 0.001
Age, years	64.6 (14.3)	73.1 (11.1)	62.1 (14.0)	< 0.001
Time from onset to hospital, hours	12 (3–48)	6 (3–24)	18.5 (4–48)	< 0.001
Comorbidities				
Hypertension	400 (51.9)	80 (61.1)	276 (50.0)	0.023
Diabetes mellitus	173 (22.4)	26 (19.8)	123 (22.3)	0.544
Hyperlipidemia	30 (3.9)	3 (2.3)	21 (3.8)	0.560
Atrial fibrillation	89 (11.5)	20 (15.3)	53 (9.6)	0.059
Current or past smoker	326 (42.3)	42 (32.1)	249 (45.1)	0.007
Current or past drinker	263 (34.1)	35 (26.7)	199 (36.1)	0.043
NIHSS score on admission	5 (2–12)	13 (8–20)	4 (2–8)	< 0.001
GCS score on admission	15 (13–15)	12 (9–15)	15 (14–15)	< 0.001
Neuroimaging				
Ischemia extent > 2/3 of MCA territory	95 (12.3)	40 (30.5)	26 (4.7)	< 0.001
Ischemia extent > 1/2 of MCA territory	121 (15.7)	55 (42.0)	34 (6.2)	< 0.001
Midline shift	72 (9.3)	34 (26.0)	18 (3.3)	< 0.001
Intravenous thrombolysis	40 (5.2)	10 (7.6)	28 (5.1)	0.250
Endovascular treatment	27 (3.5)	5 (3.8)	18 (3.3)	0.751

*Note*: Values are presented as mean (standard deviation), median (interquartile range), or number (%).

Abbreviations: GCS, Glasgow Coma Scale; MCA, middle cerebral artery; mRS, modified Ranking scale; NIHSS, National Institutes of Health Stroke Scale.

^a^Comparison between patients with mRS of 4–6 and 0–3.

Fifty patients (6.5%) were dead or were transferred to the intensive care unit during hospitalization. Eighty‐eight (11.4%) patients were lost to follow‐up at 3 months after stroke. Of the remaining 683 patients, 131 patients had poor functional outcomes (mRS 4–6) at 3 months. Compared with patients with good outcomes (mRS 0–3), patients with poor outcomes (mRS 4–6) at 3 months were older, with a mean difference of 11.0 years (95% CI, 8.8–13.2; *p* < 0.001), were less likely to be male (*p* < 0.001), smokers (*p* = 0.007) and drinkers (*p* = 0.043), had a higher proportion of hypertension (*p* = 0.023), showed a higher median NIHSS score (*p* < 0.001) and a lower GCS score (*p* < 0.001) on admission, presented with larger ischemic extents (*p* < 0.001), and had a higher prevalence of midline shift (*p* < 0.001).

### 3.3 Comparison of the Diagnostic Criteria for SIS

Based on the univariate logistic regression analysis, all diagnostic criteria were associated with poor outcomes at 3 months (Table 3). In multivariable logistic regression models adjusted for covariates that were clinically relevant or showed a *p* value < 0.20 in univariable analyses (age, sex, hypertension, atrial fibrillation, smoking status, drinking status, and time from onset to admission), all diagnostic criteria remained associated with poor outcomes at 3 months independently (adjusted OR range, 8.50–14.66; all *p* < 0.001; Table 3). No serious multicollinearity was observed in the multivariable models; the maximum variance inflation factor was 1.83, and the minimum tolerance was 0.55 across all models.

**TABLE 3:** Univariable and multivariable logistic regression of diagnostic criteria for severe ischemic stroke against poor outcomes at 3 months.

	Univariable analysis			Multivariable analysis^a^		
Diagnostic criteria	OR	95% CI	*p* value	OR	95% CI	*p* value
GCS ≤ 8	10.72	5.44–19.03	< 0.001	9.57	4.73–19.34	< 0.001
NIHSS 1a ≥ 1	8.91	5.70–13.92	< 0.001	9.21	5.54–15.28	< 0.001
NIHSS ≥ 15	11.46	7.14–18.40	< 0.001	10.43	6.14–17.70	< 0.001
NIHSS ≥ 20	10.60	5.68–19.77	< 0.001	8.50	4.28–16.89	< 0.001
Ischemic extent > 1/2 of the MCA territory	11.03	6.75–18.01	< 0.001	14.66	8.18–26.29	< 0.001
Ischemic extent > 2/3 of the MCA territory	8.89	5.17–15.28	< 0.001	10.10	5.41–18.88	< 0.001
Midline shift	10.40	5.65–19.16	< 0.001	12.79	6.22–26.30	< 0.001
Composite criteria 1	12.23	6.02–24.84	< 0.001	13.95	6.03–32.28	< 0.001
Composite criteria 2	9.78	4.85–19.73	< 0.001	10.18	4.55–22.78	< 0.001
Composite criteria 3	11.60	5.54–24.29	< 0.001	12.12	5.16–28.43	< 0.001

*Note*: Composite criteria 1: NIHSS > 15 + NIHSS 1a ≥ 1 + ischemic extent > 1/2 of the MCA territory. Composite criteria 2: NIHSS ≥ 15 + NIHSS 1a ≥ 1 + ischemic extent > 2/3 of the MCA territory. Composite criteria 3: NIHSS > 15 + GCS ≤ 13 + ischemic extent > 2/3 of the MCA territory.

Abbreviations: CI, confidence interval; GCS, Glasgow Coma Scale; MCA, middle cerebral artery; NIHSS, National Institutes of Health Stroke Scale; OR, odds ratio.

^a^Multivariable logistic regression models were adjusted for age, sex, hypertension, atrial fibrillation, smoking status, drinking status, and time from onset to admission.

Among the ten diagnostic criteria, a NIHSS score ≥15 had the maximal Youden index (0.395) and AUC (0.697, 95% CI 0.641–0.754) for predicting poor outcomes at 3 months, followed by NIHSS 1a ≥ 1 (Youden index 0.387; AUC 0.693, 95% CI 0.637–0.750) and the ischemic extent > 1/2 of the MCA territory (Youden index 0.358; AUC 0.679, 95% CI 0.621–0.737, Table 4). Three composite criteria had similar predictive performance for poor outcomes at 3 months (Composite criteria 1: Youden index 0.192; AUC 0.596, 95% CI 0.537–0.655; Composite criteria 2: Youden index 0.167; AUC 0.584, 95% CI 0.525–0.642; and Composite criteria 3: Youden index 0.171; AUC 0.585, 95% CI 0.527–0.644). Despite the poor comprehensive diagnostic ability, the composite criteria had a high specificity of over 97% and a NPV of nearly 84%. For death or transfer to the intensive care unit during hospitalization, NIHSS 1a ≥ 1 had the maximal Youden index (0.596) and AUC (0.798, 95% CI 0.728–0.869), followed by NIHSS score ≥ 15 (Youden index 0.539; AUC 0.769, 95% CI 0.692–0.847) and NIHSS score ≥ 20 (Youden index 0.492; AUC 0.746, 95% CI 0.660–0.832, Table S2). In line with the above results, three composite criteria had the lowest predictive performance for in‐hospital death or transfer to the intensive care unit (Composite criteria 1: Youden index 0.326; AUC 0.663, 95% CI 0.572–0.754; Composite criteria 2: Youden index 0.309; AUC 0.654, 95% CI 0.563–0.746; and Composite criteria 3: Youden index 0.311; AUC 0.656, 95% CI 0.564–0.747).

**TABLE 4:** Receiver operating characteristic curve analysis, sensitivity, specificity, PLR, NLR, PPV, and NPV of the diagnostic criteria in discriminating poor outcomes at 3 months.

Diagnostic criteria	AUC	95% CI	*p* value	Sensitivity (%) (95% CI)	Specificity (%) (95% CI)	Youden Index	PLR	NLR	PPV (%) (95% CI)	NPV(%) (95% CI)
GCS ≤ 8	0.607	0.548–0.666	< 0.001	24.4% (17.9%–32.4%)	96.9% (95.1%‐98.1%)	0.21	7.93	0.78	65.3% (51.3%–77.1%)	84.4% (81.4%–87.0%)
NIHSS 1a ≥ 1	0.693	0.637–0.750	< 0.001	48.1% (39.7%–56.6%)	90.6% (87.9%–92.7%)	0.39	5.11	0.57	54.8% (45.7%–63.6%)	88.0% (85.1%–90.4%)
NIHSS ≥ 15	0.697	0.641–0.754	< 0.001	46.6% (38.2%–55.1%)	92.9% (90.5%–94.8%)	0.39	6.59	0.57	61.0% (51.2%–70.0%)	88.0% (85.1%–90.4%)
NIHSS ≥ 20	0.611	0.552–0.669	< 0.001	25.2% (18.5%–33.3%)	96.9% (95.1%–98.1%)	0.22	8.18	0.77	66.0% (52.2%–77.6%)	84.5% (81.5%–87.1%)
Ischemic extent > 1/2 of the MCA territory	0.679	0.621–0.737	< 0.001	42.0% (33.9%–50.5%)	93.8% (91.5%–95.6%)	0.36	6.82	0.62	61.8% (51.4%–71.2%)	87.2% (84.3%–89.7%)
Ischemic extent > 2/3 of the MCA territory	0.629	0.570–0.688	< 0.001	30.5% (23.3%–38.9%)	95.3% (93.2%–96.8%)	0.26	6.48	0.73	60.6% (48.5%–71.5%)	85.3% (82.2%–87.8%)
Midline shift	0.613	0.555–0.672	< 0.001	26.0% (19.2%–34.1%)	96.7% (94.9%–97.9%)	0.23	7.96	0.77	65.4% (51.8%–76.8%)	84.6% (81.6%–87.2%)
Composite criteria 1	0.596	0.537–0.655	0.001	21.4% (15.2%–29.2%)	97.8% (96.2%–98.8%)	0.19	9.83	0.80	70.0% (54.6%–81.9%)	84.0% (80.9%–86.6%)
Composite criteria 2	0.584	0.525–0.642	0.003	19.1% (13.3%–26.7%)	97.6% (96.0%–98.6%)	0.17	8.10	0.83	65.8% (49.9%–78.8%)	83.6% (80.5%–86.2%)
Composite criteria 3	0.585	0.527–0.644	0.002	19.1% (13.3%–26.7%)	98.0% (96.5%–98.9%)	0.17	9.58	0.83	69.4% (53.1%–82.0%)	83.6% (80.6%–86.3%)

*Note*: Composite criteria 1: NIHSS > 15 + NIHSS 1a ≥ 1 + ischemic extent > 1/2 of the MCA territory. Composite criteria 2: NIHSS ≥ 15 + NIHSS 1a ≥ 1 + ischemic extent > 2/3 of the MCA territory. Composite criteria 3: NIHSS > 15 + GCS ≤ 13 + ischemic extent > 2/3 of the MCA territory.

Abbreviations: AUC, area under the curve; CI, confidence interval; GCS, Glasgow Coma Scale; MCA, middle cerebral artery; NIHSS, National Institutes of Health Stroke Scale; NLR, negative likelihood ratio; NPV, negative predictive value; OR, odds ratio; PLR, positive likelihood ratio; PPV, positive predictive value.

## 4 Discussion

Through the literature review, we identified 10 commonly used diagnostic criteria for SIS and compared their clinical significance by analyzing their associations with death/disability after stroke. In general, the preexisting diagnostic criteria showed modest diagnostic ability for SIS in our dataset. Among them, NIHSS score ≥ 15 and NIHSS 1a ≥ 1 had the best diagnostic power, while the composite criteria had the worst diagnostic power.

SIS accounts for up to 10% of ischemic stroke and remains a devastating disease with significant disability and mortality (Hinson et al. 2020). A growing number of studies have focused on the prophylaxis and treatments of SIS, but the diagnostic criteria for SIS vary across studies. The existing criteria for SIS mainly involve the clinical and imaging components. The 10 criteria in this study were chosen due to their simplicity and utility. As for the clinical components, the symptoms representing the large vessel occlusion are sophisticated, and their identification depends on the clinicians' experience. It is challenging to integrate complicated clinical symptoms into a unified standard to diagnose SIS. GCS and NIHSS are widely used rating tools for clinical deficits and can be assessed at the bedside with a short administration time (< 10 min) (Kwah and Diong 2014). For the imaging components, the ischemic extent can be evaluated on a routine non‐contrast CT within 5 min and be easily assessed. The quantitative measurement of infarct volume on DWI is unavailable in most community hospitals. Therefore, we included the imaging criteria based on CT in our analysis. Increasing studies use composited diagnostic criteria including both the clinical and imaging factors (Fan et al. 2021; Deng et al. 2019; Neugebauer et al. 2019). The composite criteria analyzed in our study were derived from the inclusion criteria of three large randomized clinical trials investigating the decompressive craniectomy in malignant MCA infarction (i.e., the DECIMAL, DESTINY II, and HAMLET trials) (Vahedi et al. 2007; Hofmeijer et al. 2009; Jüttler et al. 2014).

We found that NIHSS ≥ 15 and NIHSS 1a ≥ 1 had the best diagnostic ability for SIS compared to the other criteria. The NIHSS score is linearly associated with a poor outcome and a higher baseline NIHSS score is usually considered “severe stroke” (Adams et al. 1999). However, there is no definite consensus on the cut point of the NIHSS score for defining SIS recently. The cut point of 15 on NIHSS had a relatively high frequency reported in the previous studies, so we included NIHSS ≥ 15 in the final analysis and confirmed its good diagnostic ability for SIS among the existing criteria. Impaired consciousness is common in the early stage of large hemispheric stroke (Li et al. 2020). Li et al. (2020) found that impaired consciousness at stroke onset was associated with a high frequency of stroke‐related complications, especially brain edema. Both GCS and item 1a of NIHSS are used to define the impairment of consciousness in research, while NIHSS 1a is easier to apply than GCS. Our findings revealed that the NIHSS score remained the primary tool for diagnosing SIS. The three composite criteria had similar diagnostic abilities, and all of them showed low comprehensive diagnostic power for SIS. However, the specificity of the composite criteria was up to 97%, with the NPV being nearly 84%, suggesting that the composite criteria had a low false‐positive rate for diagnosing SIS. Thus, it may be reasonable to use the composite criteria in the clinical trials on aggressive treatments with high risk, such as the decompression craniectomy. Our findings may be particularly helpful for community hospitals to identify SIS at the early stage, where advanced neuroimaging or specialized stroke services may not be immediately available, because the evaluated criteria are based on practical bedside and routine clinical information.

There is no recognized gold standard for SIS worldwide. Given that the majority of patients with SIS have disastrous prognoses, we determined to use a mRS score of 4–6 at 3 months after stroke as the primary outcome. Similar to all critically ill patients, attention to airway, breathing, and hemodynamic support is the priority of SIS (Sheth 2015). Immediate and specialized neurointensive care is recommended for patients with SIS to plan close monitoring and comprehensive treatment according to the guidelines from the American Heart Association/American Stroke Association (Wijdicks et al. 2014). Thus, the death during hospitalization or transfer to the intensive care unit was set as the secondary outcome.

This study has some limitations to be noted. First, the retrospective single‐center design may have introduced selection bias and may limit the generalizability of the findings. Second, 88 patients (11.4%) were lost to follow‐up at 3 months, which may have biased the estimate of the primary outcome. Third, in‐hospital complications after SIS, such as pneumonia and stress ulcers, were the important causes of poor outcomes, while the information on complications was unavailable in our retrospective dataset. Nevertheless, we excluded patients who were complicated with severe systemic diseases on admission, which may alleviate the bias resulting from the systemic diseases. Besides, some in‐hospital treatments (i.e., endotracheal intubation, tracheotomy, and decompressive surgery) and unstable vital signs could directly reflect the severe medical condition of SIS. These detailed indicators were not included in the outcome measurements in the present study. However, transfer to the intensive care unit suggests the urgency of airway management and the maintenance of vital signs, and all patients who receive decompressive surgery enter the intensive care unit in our hospital. Thus, the existing secondary outcome in this study could reflect substantially the severe medical condition during hospitalization. Some adjusted odds ratios also had wide 95% CI, especially for stricter composite criteria and the in‐hospital severe‐outcome analysis; this imprecision may reflect the limited number of events and the low prevalence of some criteria, and these estimates should therefore be interpreted cautiously. Finally, the diagnostic ability of the criteria based on MRI was not evaluated in our cohort. Future prospective multicenter studies should further validate the reproducibility and generalizability of these criteria in broader and more heterogeneous ischemic stroke populations, including stroke related to systemic diseases such as antiphospholipid syndrome. Such studies should also explore whether advanced MRI features, blood‐based biomarkers, and AI‐assisted imaging or clinical prediction models can improve the accuracy and consistency of SIS diagnosis.

## 5 Conclusions

We performed an objective comparison of the commonly used diagnostic criteria for SIS in this study. The NIHSS score remained the primary tool to diagnose SIS. The severity of stroke assessed by NIHSS ≥ 15 and the impaired consciousness defined by NIHSS 1a ≥ 1 showed the best diagnostic ability. Our results provide scientific evidence to identify a simple and unified diagnostic criterion for SIS, which will help to minimize inter‐observer variation in the studies of SIS, enable cross‐comparison between research groups, and facilitate communication of case series in clinical settings.

## Author Contributions

**Xiaoyu Hua**: literature search, investigation, methodology, formal analysis, writing – original draft. **Jinzhen Song**: investigation, data collection, software, writing – review. **Chengfeng Xing**: investigation, data collection, software, writing – review. **Chenchen Wei**: conceptualization, supervision, writing – review and editing.

## Funding

This study was supported by National Natural Science Foundation of China (Grant No.82401545 and No. 82402287) and Shandong Provincial Natural Science Foundation (Grant No.ZR2022QH363 and No.ZR2022QH310).

## Ethics Statement

The project was approved by the Biomedical Research Ethics Committee of the Affiliated Hospital of Qingdao University (No. QDFYWZLL‐20240114‐003), and the protocol conformed to local ethics criteria for human research. All participants provided the written informed consent.

## Conflicts of Interest

The authors declare no conflicts of interest.

## Supporting information




**Supplemental Figure S1** Flowchart of patient inclusion and follow‐up.
**Supplemental Figure S2** Receiver operating characteristic curves of diagnostic criteria for poor functional outcome at 3 months.
**Supplemental Figure S3** Receiver operating characteristic curves of diagnostic criteria for in−hospital death or transfer to the intensive care unit.
**Supplemental Table S1** Inclusion/exclusion criteria for the study participants.
**Supplemental Table S2** Receiver operating characteristic curve analysis and diagnostic performance of the criteria for in‐hospital death or transfer to the intensive care unit.
STARD 2015 checklist

## Data Availability

The data supporting the findings of this study are available on reasonable request from the corresponding author.
